# Ultrasound findings suggestive of microscopic extranodal extension in papillary thyroid carcinoma

**DOI:** 10.1007/s10396-025-01573-w

**Published:** 2025-09-20

**Authors:** Noriko Miyamoto, Mitsuyoshi Hirokawa, Miyoko Higuchi, Maki Oshita, Makoto Kawakami, Hiroyuki Yamaoka, Makoto Fujishima, Akira Miyauchi, Takashi Akamizu

**Affiliations:** 1 Department of Clinical Laboratory, Kuma Hospital, 8-2-35 Shimoyamate-Dori, Chuo-Ku, Kobe, Hyogo 650-0011 Japan; 2 Department of Diagnostic Pathology and Cytology, Kuma Hospital, 8-2-35 Shimoyamate-Dori, Chuo-Ku, Kobe, Hyogo 650-0011 Japan; 3 Medical Information Management Section, Kuma Hospital, 8-2-35 Shimoyamate-Dori, Chuo-Ku, Kobe, Hyogo 650-0011 Japan; 4 Department of Internal Medicine, Kuma Hospital, 8-2-35 Shimoyamate-Dori, Chuo-Ku, Kobe, Hyogo 650-0011 Japan; 5 Department of Surgery, Kuma Hospital, 8-2-35 Shimoyamate-Dori, Chuo-Ku, Kobe, Hyogo 650-0011 Japan

**Keywords:** Ultrasound, Papillary thyroid carcinoma, Lymph-node metastasis, Extranodal extension, Node matting

## Abstract

**Purpose:**

Extranodal extension (ENE) of metastatic carcinoma in patients with papillary thyroid carcinoma (PTC) has been associated with an increased risk of recurrent disease, persistent disease, and disease-specific mortality; however, ultrasound findings suggestive of ENE have not been well established. In this study, we aimed to identify ultrasound findings suggestive of microscopic ENE and validate them histologically.

**Methods:**

We retrospectively examined the ultrasound and histological findings of 21 PTC patients with microscopic ENE and 46 without ENE.

**Results:**

Node matting, irregular shapes, ill-defined jagged border, and perinodal hyperechoic rims were observed in 38.1%, 57.1%, 42.9%, and 57.1% of lymph nodes with ENE, respectively, and the frequencies were significantly higher than those without ENE, with *p* values less than 0.05, 0.0005, 0.0001, and 0.0001, respectively. The sensitivity and specificity of cases with any one of irregular shapes, ill-defined jagged border, and perinodal hyperechoic rims were 81.0% and 82.6%, respectively. Histologically, node matting, irregular shape, ill-defined jagged border, and a perinodal hyperechoic rim correspond to adhesion between lymph nodes, extensive invasion, minimal invasion, and invasion into adipose tissue, respectively.

**Conclusions:**

We would argue that any irregular shape, ill-defined jagged border, and perinodal hyperechoic rim can be accepted as findings indicative of microscopic ENE.

## Introduction

The American Thyroid Association guidelines proposed a stratification system for the risk of recurrence in patients with well-differentiated thyroid carcinoma based on staging, histological type, vascular invasion, lymph-node metastasis, and distant metastasis [1]. For lymph-node metastasis, the number (≦5 or > 5) and size (< 0.2 cm, < 3 cm, or ≧5 cm) of involved lymph nodes were adopted as parameters. Extranodal extension (ENE) of metastatic papillary thyroid carcinoma (PTC) is reportedly associated with an increased risk of recurrent disease, persistent disease, and disease-specific mortality [2–11]. ENE was assigned pathologically by tumor cells extending beyond the lymph-node capsule into the perinodal fibroadipose tissue and identified by gross or microscopic examination [3, 7–9, 12]. Gross ENE is defined as lymph-node metastasis invading adjacent organs, such as the recurrent laryngeal nerve, thoracic duct, jugular vein, trachea, and esophagus [13]. Microscopic (minimal) ENE was not associated with the invasion of adjacent organs [14]. Roh et al. demonstrated that patients with microscopic and macroscopic ENE had a 2- and 3.4-fold higher risk of recurrence, respectively, than those without ENE [15]. Preoperative recognition of ENE is invaluable for clinical management.

The overall rate of ENE in patients with PTC reportedly ranges between 22 and 45% [14]; however, estimating the exact incidence remains challenging. Ultrasound is a commonly used imaging modality for evaluating cervical lymph nodes. To date, a few reports have been published on the ultrasound images of ENE in patients with PTC [16, 17]. Accumulated evidence indicates that node matting, microcalcification, cystic area > 50%, long/tall ratio < 2.0, larger diameter, and perinodal edema are ultrasound findings associated with ENE. However, these findings are yet to be fully elucidated. In this study, we aimed to identify ultrasound findings suggestive of microscopic ENE and to validate them histologically.

## Materials and methods

### Study population

We reviewed the surgical pathology data of 1197 patients with PTC who underwent thyroidectomy and lymph-node dissection at Kuma Hospital between August 2022 and July 2024, subsequently selecting 825 patients with lymph-node metastasis. Among these, 97 (11.8%) patients exhibited ENE on microscopic examination. ENE was defined as the external invasion of papillary carcinoma cells beyond the lymph-node capsule. Among the remaining 728 patients, metastatic lesions were restricted to the lymph nodes. Twenty-one patients (21.6%) in whom lymph nodes with ENE could be identified with ultrasound were included in this study. Written informed consent was obtained from the patients for the publication of this study and use of ultrasound images. Considering patients who had multiple lymph nodes with ENE, the largest lymph node was selected for the study. All lymph nodes selected for the study were lateral and showed microscopic ENE. As control subjects, we selected 46 PTC patients with lateral lymph-node metastasis but without ENE among 211 consecutive PTC patients who had undergone lymph-node dissection between January and July 2023.

### Ultrasound and data collection

Ultrasound examinations were performed using an Aplio i800 TUS-AI800 (Canon Medical Systems Co., Ltd., Otawara, Japan) or Aplio 500 TUS-A500 (Canon Medical Systems) with a PLT-1005BT probe (5–14 MHz, Canon Medical Systems) or PLI-1205BX probe (5–18 MHz, Canon Medical Systems). Clinical data were obtained from the patient’s medical records at Kuma Hospital. We retrospectively examined ultrasound reports and images saved in medical records. Ultrasound examinations were performed by 19 ultrasound technicians specializing in thyroid ultrasonography. In the case of debatable findings, clinical technologists reported the results after consulting a senior technologist. The ultrasound findings examined included lymph-node size, node matting, depth/width ratio, irregular shape, ill-defined jagged border, perinodal hyperechoic rim, punctate echogenic foci, cystic changes, and increased vascular signals. Blood flow signals were evaluated using power Doppler and/or advanced dynamic flow imaging. Lymph-node size was measured in three directions, and the largest diameter was used as the lymph-node size. Histological examinations were performed using representative hematoxylin and eosin-stained preparations.

### Statistical analysis

Statistical analysis was performed using Fisher’s exact tests, Welch's t test, and a *p* value of < 0.05 was considered a statistically significant difference.

## Results

**Fig. 1:**
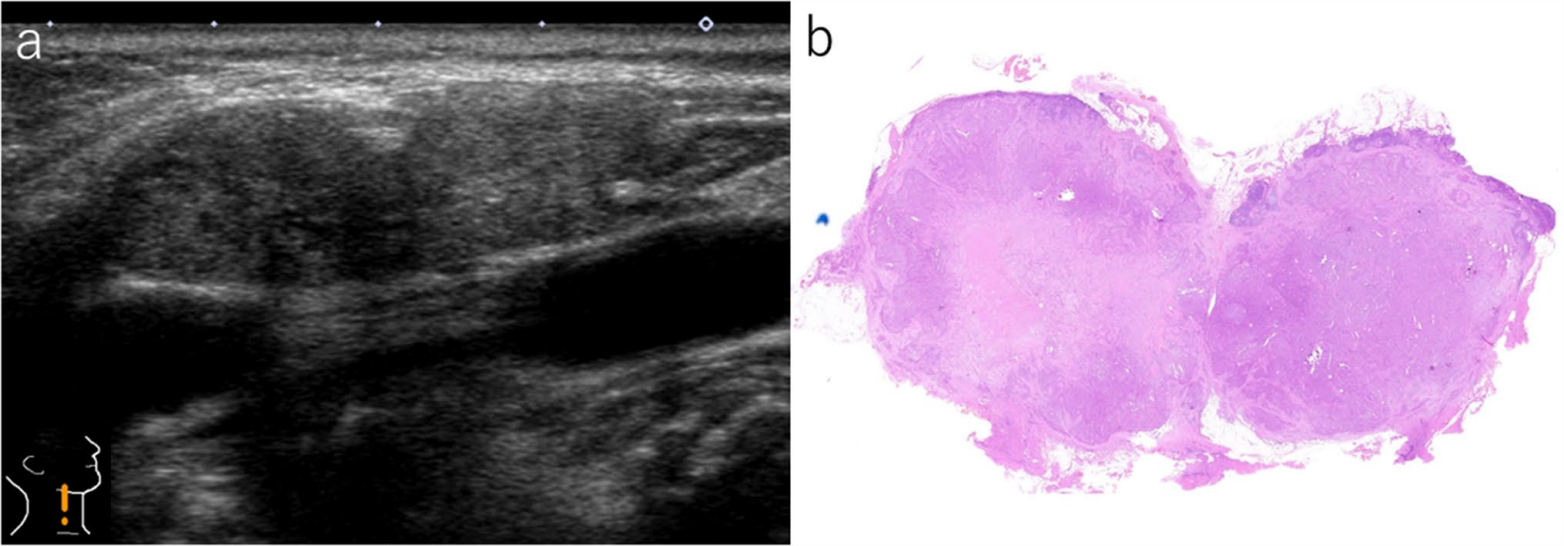
Lymph nodes with extranodal extension. **a** Two adjacent lymph nodes with ill-defined jagged border show node matting (B-mode, longitudinal scan). **b** The lymph nodes adhere to each other via extranodal extension (gross image of hematoxylin and eosin-stained preparation)

**Fig. 2:**
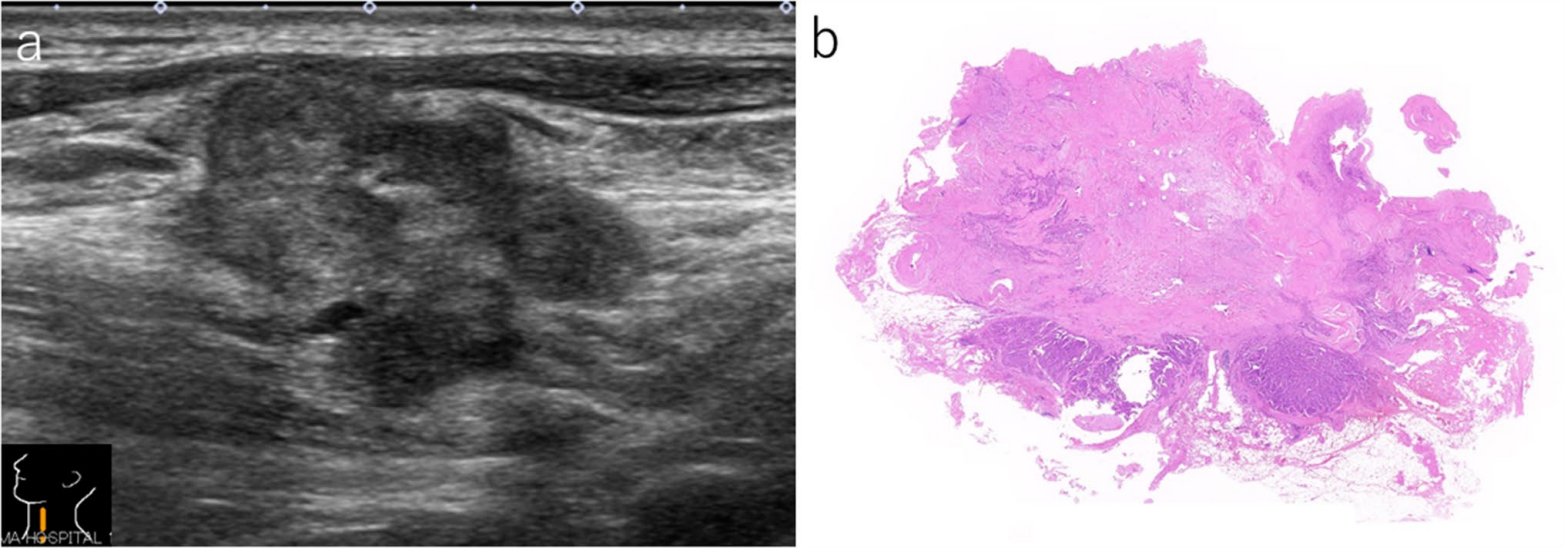
Lymph node with extensive extranodal extension. **a** Irregular-shaped and lobulated lymph node shows heterogeneous internal echogenicity (B-mode, longitudinal scan). **b** Carcinoma cells extensively invade the surrounding tissue (gross image of hematoxylin and eosin-stained preparation)

**Fig. 3:**
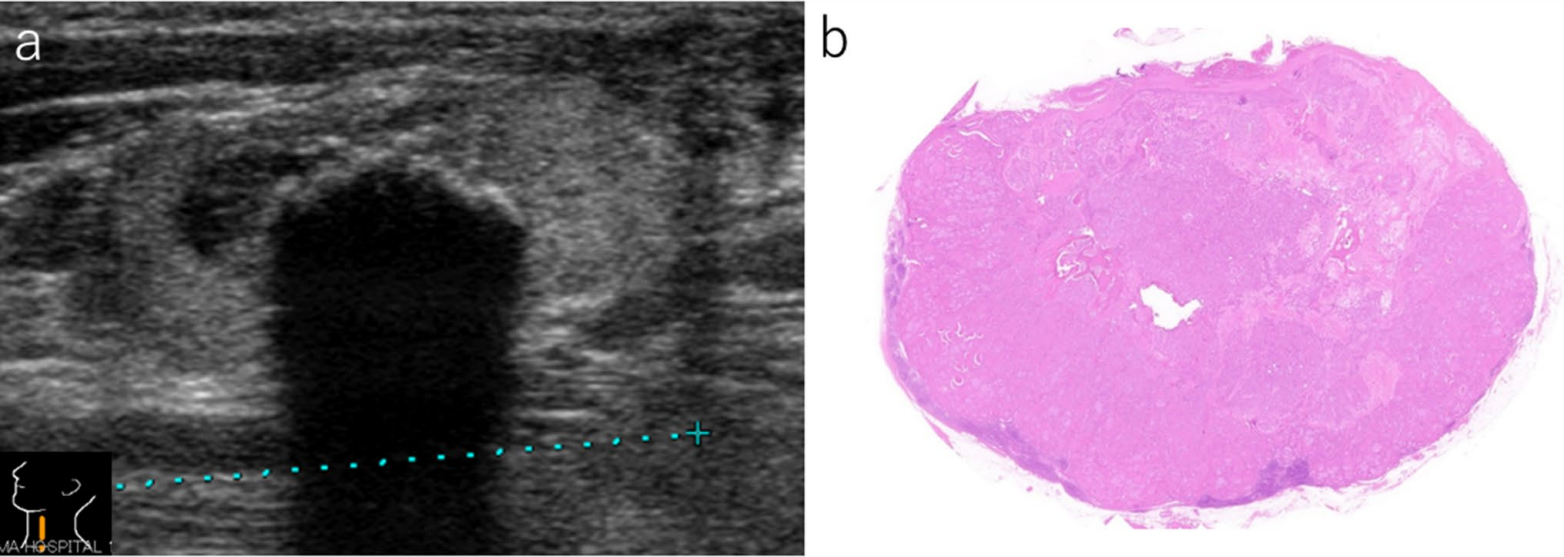
Lymph node with minimal extranodal extension. **a** The border is ill-defined and jagged. Internal calcification can be observed (B-mode, longitudinal scan). **b** Carcinoma cells show minimal invasion into the surrounding tissue (gross image of hematoxylin and eosin-stained preparation)

**Fig. 4:**
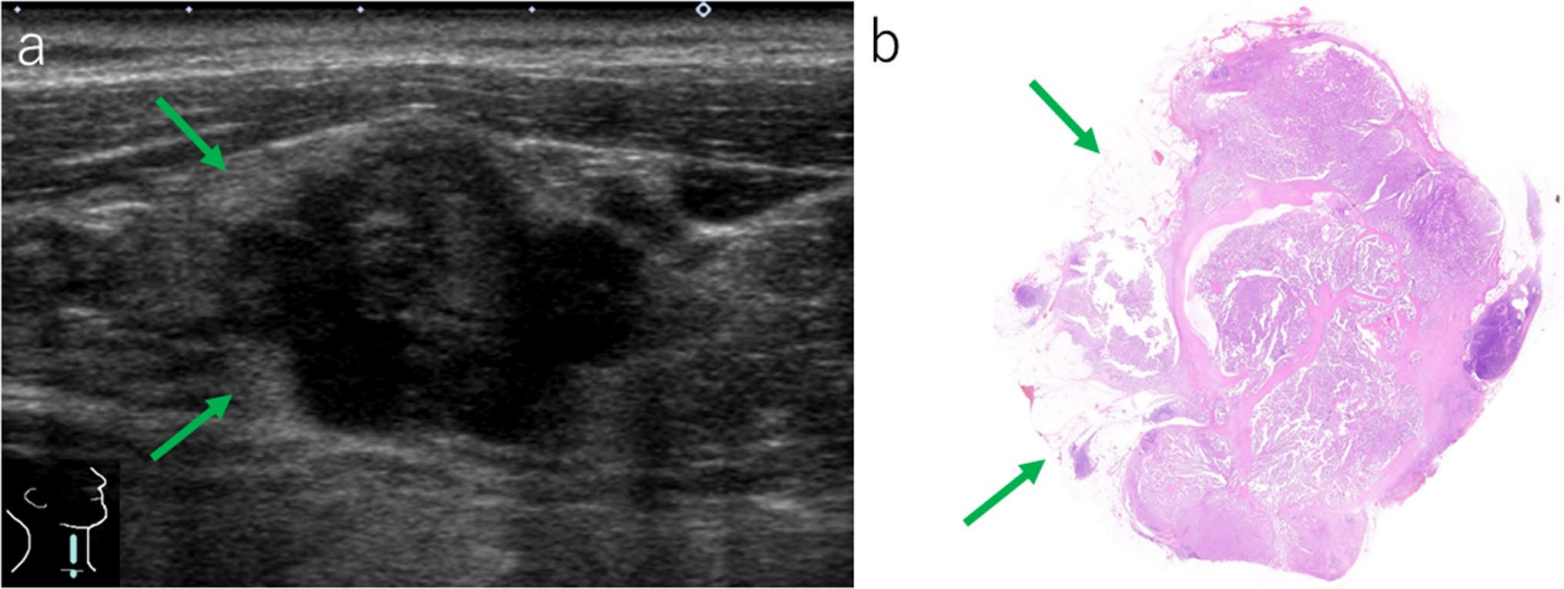
Lymph node with extranodal extension. **a** Irregular-shaped lymph node shows an ill-defined border. Perinodal hyperechoic rim (green arrows) can be observed (B-mode, longitudinal scan). **b** The area corresponding to the perinodal hyperechoic rim comprises a mixture of adipose tissue and invasive carcinoma cells (gross image of hematoxylin and eosin-stained preparation)

Table 1 presents the ultrasound findings of nodal metastases with and without ENE in patients with PTC. Lymph nodes with ENE were larger than those without ENE, although no statistically significant differences were detected. Examining two enlarged lymph nodes located adjacent to each other, node matting (Fig. 1a) was observed in 38.1% of the lymph nodes with ENE, and the frequency was significantly higher than that without ENE (*p* < 0.05). Upon histological examination, we found that two lymph nodes exhibiting node matting were predominantly occupied by metastatic carcinoma cells and adhered to each other via the ENE (Fig. 1b). A depth/width ratio > 0.5 was detected in most lymph nodes with and without ENE. No significant differences were found in the taller-than-wide shapes. An irregular shape (Fig. 2a) was observed in 57.1% and 13.0% of lymph nodes with and without ENE, respectively, and the difference was statistically significant (*p* < 0.0005). Histologically, irregularly shaped lymph nodes showed extensive invasion of the surrounding tissues (Fig. 2b). Ill-defined jagged borders (Fig. 3a) were observed in 42.9% of lymph nodes with ENE, but not in those without ENE. The finding had 100% specificity, with *p* values of 0.0001. In lymph nodes with an ill-defined jagged border, invasion into the surrounding tissue was minimal (Fig. 3b). A perinodal hyperechoic rim (Fig. 4a) was detected in 57.1% and 6.5% of lymph nodes with and without ENE, respectively (*p* < 0.0001). The area corresponding to a perinodal hyperechoic rim comprised a mixture of adipose tissue and invasive carcinoma cells (Fig. 4b) with no edematous changes. Frequent punctate echogenic foci, loss of fatty hilum, > 50.0% cystic change, and increased vascular signals were non-significant. Pure solid composition was more frequently seen in lymph nodes with ENE (85.7%) than those without ENE (39.1%) (*p* < 0.0005).

**Table 1:** Ultrasound findings of nodal metastasis with and without extranodal extension in patients with papillary thyroid carcinoma

	Extranodal extension		*p *value
	Present (*n* = 21)	Absent (*n* = 46)	
Lymph-node size (mean)	6–50 mm (22.3)	6–44 mm (18.9)	NS
Lymph-node size > 30 mm	3 (14.3%)	3 (6.5%)	NS
Node matting	8 (38.1%)	6 (13.0%)	< 0.05
Depth/width ratio > 0.5	18 (85.7%)	29 (63.0%)	NS
Irregular shape	12 (57.1%)	6 (13.0%)	< 0.0005
Ill-defined jagged border	9 (42.9%)	0 (0%)	< 0.0001
Perinodal hyperechoic rim	12 (57.1%)	3 (6.5%)	< 0.0001
Loss of fatty hilum	21 (100%)	44 (95.7%)	NS
Punctate echogenic foci	6 (28.6%)	9 (19.6%)	NS
Cystic area > 50.0%	0 (0%)	6 (13.0%)	NS
Pure solid composition	18 (85.7%)	18 (39.1%)	< 0.0005
Increased vascular signal	14 (66.7%)	24 (52.2%)	NS

*NS* not significant

Table 2 shows the diagnostic accuracy of ENE based on three findings with strong significant differences: irregular shape, ill-defined jagged border, and perinodal hyperechoic rim. The specificity and positive predictive value in cases with all three findings were 100%, but the sensitivity was low (23.8%). The sensitivity and specificity of cases with any one of those findings were 81.0% and 82.6%, respectively.

**Table 2:** Diagnostic accuracy of extranodal extension based on three findings: irregular shape, ill-defined jagged border, and perinodal hyperechoic rim

Number of findings	Sensitivity	Specificity	Positive predictive value	Negative predictive value
3	23.8% (5/21)	100% (46/46)	100% (5/5)	74.2% (46/62)
2 or 3	42.9% (9/21)	97.8% (45/46)	90.0% (9/10)	67.2% (45/67)
1 or more	81.0% (17/21)	82.6% (38/46)	68.0% (17/25)	90.5% (38/42)

## Discussion

ENE—defined as invasion beyond the lymph-node capsule of carcinoma cells that have metastasized into the lymph nodes—is divided into gross and microscopic ENE [2, 7–9, 12–14]. Both ENE types are associated with a higher risk of recurrence than lymph nodes without ENE [15]. In 2016, Qualliotine et al. first analyzed the ultrasound findings of ENE in PTC, noting associations with node matting, cystic content > 50%, unclear margins, and perinodal edema [17]. In 2018, Mu et al. reported higher incidences of node matting, microcalcification, cystic area, long/tall ratio < 2.0, and larger diameters in lymph nodes with ENE than in those without ENE [16].

In the present study, we focused on the ultrasound findings of microscopic ENE. Lymph-node size > 30 mm, depth/width ratio > 0.5, loss of fatty hilum, punctate echogenic foci, and increased vascular signal were more frequently observed in lymph nodes with ENE than in those without ENE; however, no statistically significant differences were detected. We think that those findings may be suggestive of ENE, but their significance is limited, and they were indicative of nodal metastasis.

Qualliotine et al. reported cystic content > 50% as one of the ultrasound findings of lymph nodes with ENE [17]. However, in the present study, the finding was not observed in lymph nodes with ENE. A pure solid composition, which means no cystic area, was observed at a significantly higher frequency in lymph nodes with ENE. In the thyroid, cystic PTC has a favorable prognosis for its size [18]. Similarly, in the lymph nodes, metastatic lesions with cystic components are likely to be more indolent. However, we have no basis for asserting that the pure solid composition itself is associated with ENE.

Node matting refers to the obscurity of the border between two or more adjacent lymph nodes. It was introduced as an ultrasound finding suggesting ENE of the axillary lymph nodes in patients with breast cancer [19]. Node matting has also been reported in the ENE of PTC, with incidences of 13.3% [16] and 45.5% [17]. In the present study, node matting was detected in 38.1% of lymph nodes with ENE. Histological examination suggested adhesion of the adjacent lymph nodes due to ENE. However, we also observed node matting in lymph nodes without ENE, albeit less frequently. It is impossible to determine whether the finding would appear even in cases without ENE, or whether it is a false positive from only observing a few images. To distinguish between node matting with and without ENE, it is crucial to determine whether the two lymph nodes are fixed during real-time sonography; however, this could not be examined owing to the retrospective nature of the current study. Nevertheless, node matting is a valuable finding indicating the presence of ENE.

Qualliotine et al. reported an ill-defined border in 38% of lymph nodes with ENE, with a specificity of 61.1% [17]. In contrast, Mu et al. found that an ill-defined border was present at an equal frequency in lymph nodes with and without ENE [16]. In the present study, an ill-defined jagged border was observed in lymph nodes with ENE but not in those without ENE; the specificity was 100%. We conclude that both findings are characteristic of ENE, and correspond to the early stages of ENE. However, it is important to acknowledge that this is not always the case as the prevalence was not particularly high (33.3–42.9%).

A hyperechoic halo encircling the lymph nodes has been described as perinodal edema suggestive of ENE [19]. Qualliotine et al. reported perinodal edema in 18.2% of lymph nodes with ENE but not in those without ENE [17]. We also observed a perinodal hyperechoic rim in 57.1% of lymph nodes with ENE, and the frequency was significantly higher than that in lymph nodes without ENE. We confirmed that the presence of a perinodal hyperechoic rim strongly suggested ENE. However, we believe that the term perinodal edema is inappropriate. Perinodal edema was not detected in lymph nodes with perinodal hyperechoic rims. This finding corresponded to a mixture of adipose tissue and invasive PTC cells.

We examined the diagnostic accuracy of ENE based on three findings with strong significant differences: irregular shape, ill-defined jagged border, and perinodal hyperechoic rim. The sensitivity and specificity of cases with any one of them were 81.0% and 82.6%, respectively. Therefore, we would argue that any one of irregular shape, ill-defined jagged border, and perinodal hyperechoic rim can be accepted as a finding suggestive of microscopic ENE.

This study had several limitations. The study was retrospective in nature and was performed using saved images. If video data were available, a more detailed study could have been conducted. The detection rate of lymph nodes with ENE was low. Cases with central lymph nodes were not included in this study. It suggests that the evaluation of ENE in central lymph nodes is definitely difficult. The ability of the sonographer and the quality of the ultrasound equipment must have also affected the results of detailed findings.

## Conclusion

We would argue that any one of irregular shape, ill-defined jagged border, and perinodal hyperechoic rim can be accepted as a finding suggestive of microscopic ENE. Histologically, these findings corresponded to extensive invasion, minimal invasion, and invasion into adipose tissue. Taken together, our results may be useful in determining preoperative treatment decisions and prognostic indicators.

## Data Availability

The data supporting the findings of this study are available from the corresponding author upon reasonable request.
