# Correction: Ultrasound findings suggestive of microscopic extranodal extension in papillary thyroid carcinoma

**DOI:** 10.1007/s10396-025-01594-5

**Published:** 2025-11-01

**Authors:** Noriko Miyamoto, Mitsuyoshi Hirokawa, Miyoko Higuchi, Maki Oshita, Makoto Kawakami, Hiroyuki Yamaoka, Makoto Fujishima, Akira Miyauchi, Takashi Akamizu

**Affiliations:** 1 Department of Clinical Laboratory, Kuma Hospital, 8-2-35 Shimoyamate-Dori, Chuo-Ku, Kobe, Hyogo 650-0011 Japan; 2 Department of Diagnostic Pathology and Cytology, Kuma Hospital, 8-2-35 Shimoyamate-Dori, Chuo-Ku, Kobe, Hyogo 650-0011 Japan; 3 Medical Information Management Section, Kuma Hospital, 8-2-35 Shimoyamate-Dori, Chuo-Ku, Kobe, Hyogo 650-0011 Japan; 4 Department of Internal Medicine, Kuma Hospital, 8-2-35 Shimoyamate-Dori, Chuo-Ku, Kobe, Hyogo 650-0011 Japan; 5 Department of Surgery, Kuma Hospital, 8-2-35 Shimoyamate-Dori, Chuo-Ku, Kobe, Hyogo 650-0011 Japan

**Correction: Journal of Medical Ultrasonics** 10.1007/s10396-025-01573-w

In the sentence beginning ‘In the present study, node matting was detected…..’ in this article, the value ‘40.0%’ should have read ‘38.1%’.

In the sentence beginning ‘Node matting, irregular shapes, ill-defined jagged border…..’ in this article, the value ‘40.0%’ should have read ‘38.1%’.

In the sentence beginning ‘Examining two enlarged lymph nodes located adjacent to each other…..’ in this article, the value ‘40.0%’ should have read ‘38.1%’.

In Table 1 of this article, the data in the third row of the second column was incorrectly stated as “8 (40.0%)” and should have been “8 (38.1%)”.

In Table 1 of this article, the data in the ninth row of the third column was incorrectly stated as “9 (19.1%)” and should have been “9 (19.6%)”. The incorrect and the corrected versions of Table 1 are given below.

Incorrect Table:

**Table 1:** Ultrasound findings of nodal metastasis with and without extranodal extension in patients with papillary thyroid carcinoma

	Extranodal extension		*p *value
	Present (*n* = 21)	Absent (*n* = 46)	
Lymph-node size (mean)	6–50 mm (22.3)	6–44 mm (18.9)	NS
Lymph-node size > 30 mm	3 (14.3%)	3 (6.5%)	NS
Node matting	8 (40.0%)	6 (13.0%)	< 0.05
Depth/width ratio > 0.5	18 (85.7%)	29 (63.0%)	NS
Irregular shape	12 (57.1%)	6 (13.0%)	< 0.0005
Ill-defined jagged border	9 (42.9%)	0 (0%)	< 0.0001
Perinodal hyperechoic rim	12 (57.1%)	3 (6.5%)	< 0.0001
Loss of fatty hilum	21 (100%)	44 (95.7%)	NS
Punctate echogenic foci	6 (28.6%)	9 (19.1%)	NS
Cystic area > 50.0%	0 (0%)	6 (13.0%)	NS
Pure solid composition	18 (85.7%)	18 (39.1%)	< 0.0005
Increased vascular signal	14 (66.7%)	24 (52.2%)	NS

*NS* not significant

Corrected Table:

**Table 1:** Ultrasound findings of nodal metastasis with and without extranodal extension in patients with papillary thyroid carcinoma

	Extranodal extension		*p *value
	Present (*n* = 21)	Absent (*n* = 46)	
Lymph-node size (mean)	6–50 mm (22.3)	6–44 mm (18.9)	NS
Lymph-node size > 30 mm	3 (14.3%)	3 (6.5%)	NS
Node matting	8 (38.1%)	6 (13.0%)	< 0.05
Depth/width ratio > 0.5	18 (85.7%)	29 (63.0%)	NS
Irregular shape	12 (57.1%)	6 (13.0%)	< 0.0005
Ill-defined jagged border	9 (42.9%)	0 (0%)	< 0.0001
Perinodal hyperechoic rim	12 (57.1%)	3 (6.5%)	< 0.0001
Loss of fatty hilum	21 (100%)	44 (95.7%)	NS
Punctate echogenic foci	6 (28.6%)	9 (19.6%)	NS
Cystic area > 50.0%	0 (0%)	6 (13.0%)	NS
Pure solid composition	18 (85.7%)	18 (39.1%)	< 0.0005
Increased vascular signal	14 (66.7%)	24 (52.2%)	NS

*NS* not significant

The original article has been corrected.

